# Appropriate antibiotic use for patients with complicated urinary tract infections in 38 Dutch Hospital Departments: a retrospective study of variation and determinants

**DOI:** 10.1186/s12879-015-1257-5

**Published:** 2015-11-09

**Authors:** V. Spoorenberg, S. E. Geerlings, R. B. Geskus, T. M. de Reijke, J. M. Prins, M. E. J. L. Hulscher

**Affiliations:** Department of Internal Medicine, Division of Infectious Diseases, Centre for Infection and Immunity Amsterdam, Academic Medical Centre, Amsterdam, The Netherlands; Department of Clinical Epidemiology, Biostatistics and Bioinformatics, Academic Medical Centre, Amsterdam, The Netherlands; Department of Urology, Academic Medical Centre, Amsterdam, The Netherlands; Scientific Institute for Quality of Healthcare, Radboud University Nijmegen Medical Centre, Nijmegen, The Netherlands

**Keywords:** Antibiotic use, Urinary tract infection, Quality indicator, Guideline adherence, Antibiotic stewardship, Determinants

## Abstract

**Background:**

Appropriate antibiotic use in patients with complicated urinary tract infections can be measured by a valid set of nine quality indicators (QIs). We evaluated the performance of these QIs in a national setting and investigated which determinants influenced appropriate antibiotic use. For the latter, we distinguished patient, department and hospital characteristics, including organizational interventions aimed at improving the quality of antibiotic use (antibiotic stewardship elements).

**Methods:**

A retrospective, observational multicentre study included 1964 patients (58 % male sex) with a complicated urinary tract infection treated at Internal Medicine and Urology departments of 19 Dutch university and non-university hospitals. Data of 50 patients per department were extracted from medical charts. QI performance scores were calculated using previously constructed algorithms. Department and hospital characteristics were collected using questionnaires filled in by an internal medicine physician and an urologist. Regression analysis was performed to identify determinants of QI performance. Clustering at department and hospital level was taken into account through inclusion of random effects in a multi-level model.

**Results:**

Median QI performance of departments varied between 31 % (‘Treat urinary tract infection in men according to local guideline’) and 77 % (‘Perform urine culture’). The patient characteristics non-febrile urinary tract infection, female sex and presence of a urinary catheter were negatively associated with performance on many QIs. The presence of an infectious diseases physician and an antibiotic formulary were positively associated with ‘Prescribe empirical therapy according to guideline’. No other department or hospital characteristics, including stewardship elements, were consistently associated with better QI performance.

**Conclusions:**

A large inter-department variation was demonstrated in the appropriateness of antibiotic use. In particular certain patient characteristics (more than department or hospital characteristics) influenced the quality of antibiotic use. Some, but not all antibiotic stewardship elements did translate into better QI performance.

**Electronic supplementary material:**

The online version of this article (doi:10.1186/s12879-015-1257-5) contains supplementary material, which is available to authorized users.

## Background

Appropriate antibiotic use in patients with infections is important for optimal clinical outcome, to avoid preventable complications such as renal failure and *Clostridium difficile* infection, to control the growth of antibiotic resistance and to contain costs [1–3].

However, according to medical literature, up to 50 % of hospital antibiotic use is inappropriate [4, 5], and Antibiotic Stewardship Programs have been recommended to improve appropriate antibiotic use [6]. They can be considered as a menu of interventions that can be designed and adapted to fit the infrastructure of any hospital [7]. However, to successfully design effective and targeted interventions to improve antibiotic prescribing, it is first necessary to better understand the factors that influence appropriate prescribing [8, 9]. Various determinants are known to be of influence, resulting in large differences in appropriate antibiotic use between hospitals [10].

Urinary Tract Infections (UTIs) are among the most prevalent infectious diseases in the in- and outpatient setting, being a major cause of morbidity and mortality, and resulting in many hospitalizations [11]. Appropriate antibiotic use for patients with a complicated UTI was previously defined with a valid set of nine guideline-based quality indicators [12]. The objective of the current study was to assess in a large group of hospitals the performance on these nine quality indicators and to identify which determinants influenced appropriate antibiotic use. For the latter, we distinguished patient, department and hospital characteristics, including organizational interventions aimed at improving the quality of antibiotic use (stewardship elements).

## Methods

### Setting and population

Our study presents the baseline results of a cluster randomized controlled trial testing a multifaceted stewardship program to improve the appropriateness of antibiotic use in patients with a complicated UTI in hospitals (http://www.trialregister.nl; NTR1742).

Appropriateness of antibiotic use in patients with a complicated UTI was assessed at the internal medicine and urology departments of 19 university, teaching and non-teaching hospitals located throughout the Netherlands. Included were adult (≥16 years) inpatients/outpatients diagnosed in 2008 by an internist or a urologist with a complicated UTI as main diagnosis, and treated as such. We defined a complicated UTI as a UTI with one (or more) of the following characteristics: male gender, pregnancy, any functional or anatomical abnormality of the urinary tract, immunocompromising disease or medication, or a UTI with symptoms of tissue invasion or systemic infection [13]. The identification of patients as performed using the national diagnosis registration system. Subsequent manual screening took place, with the use of medical and nursing records and admission sheets. A minimum number of 50 patients per department was included. If required to reach a sufficient number also patients from 2007 were included. Excluded were patient groups for whom the Dutch national guideline does not provide a treatment recommendation (i.e. patients with a nephrostomy) and patients who were currently being treated for another infection or had been transferred from or to another hospital.

The medical ethical committee of the Academic Medical Centre Amsterdam considered our study and concluded that it was deemed exempt from their approval (ref 08.17.1775). No informed consent was obtained from patients because no interventions at the patient level were done and patient data were analysed in a retrospective design anonymously, for the aim to improve quality or healthcare.

### Variables and data collection

#### Quality indicators for complicated UTI care (dependent variables)

Between February and November 2009 the study researcher (VS) and a trained research assistant retrospectively collected data from medical and nursing charts, admission sheets, medication charts, and laboratory and culture results. The appropriateness of antibiotic use was scored using quality indicators (QIs) based on the treatment recommendations from the Dutch national guideline for the antibiotic treatment of complicated UTIs [14]. A 3-step modified Delphi approach among experts was used to systematically develop a set of nine QIs, which was subsequently validated [15] (Table 1). The evidence-based Dutch guideline comprises a general treatment recommendation for patients with complicated UTIs, as well as recommendations for subpopulations with special conditions, e.g. patients with a urinary catheter and men with chronic prostatitis. All subpopulations were included in each QI and evaluated by their own treatment recommendation. Men with a UTI scored also on an additional QI (‘Treat UTI in men according to the guideline’). This QI applies to (denominator) all men with a UTI, including those with a chronic prostatitis, but except those with a urinary catheter. It evaluates (numerator) whether they were treated in accordance with the guideline regarding the empirical therapy and treatment duration for complicated UTIs and, in case of a chronic prostatitis, whether they were treated with culture-guided therapy for the recommended duration.

**Table 1:** Set of Quality Indicators [12]

	Quality indicators
1	Perform a urine culture
2A	Prescribe empirical therapy in accordance with the national guideline
2B	Prescribe empirical therapy in accordance with the local guideline
3	Switch from intravenous to oral therapy within 72 h on the basis of the clinical condition^*^
4	Tailor antibiotic treatment on the basis of culture results
5	Use fluoroquinolones selectively (oral therapy, or in case of anaphylaxis to β-lactam antibiotics)
6A	Duration of antibiotic therapy should be at least 10 days (in accordance with the national guideline)
6B	Duration of antibiotic therapy should be according to the local guideline
7A	Treat UTI in men in accordance with the national guideline
7B	Treat UTI in men in accordance with the local guideline
8	Replace catheter after initiation of antibiotic treatment
9	Adapt antibiotic dose according to renal function

^*^Fulfilling the criteria for a safe early switch: haemodynamic stability, no gastrointestinal problems at 48 h after admission, no *Staphylococcus aureus* in the blood culture and the availability of an adequate oral antibiotic, based on culture result, or the availability of oral equivalent of the i.v. antibiotic [33]

Although local hospital antibiotic treatment guidelines are usually based on the national guideline [14], they can differ, e.g. because of local resistance patterns. Therefore, the QIs concerning prescribing empirical therapy, duration of treatment and treatment of men were scored according to the national guideline as well as according to the local guideline if available.

Previously constructed algorithms, in which data directly originating from the patient chart had to be inserted, were used to calculate the scores for each of the QIs.

#### Determinants at the patient-, department- and hospital level (independent variables)

Based on prior research findings the following potentially relevant patient, department and hospital characteristics were selected (Tables 2, 3 and 4) [9, 10, 16–20].

**Table 2:** Patient characteristics. When not all eligible patients were included (due to missing values) the number of included patients is indicated in the upper row of the cell

Patients	Internal Medicine *(n = 981)*	Urology *(n = 983)*	Total *(n = 1,964)*
Male sex^*^	381 (40)	753 (77)	1134 (58)
Age in years, median (IQR)	74 (27)	58 (28)	65 (31)
Comorbidity	*n = 981*	*n = 981*	*n = 1962*
Any	649 (66)	388 (40)	1037 (53)
Diabetes mellitus	282 (29)	99 (10)	381 (19)
Urological comorbidity and/or kidney disease	275 (28)	243 (25)	518 (26)
Urinary catheter	168 (17)	104 (11)	272 (14)
Glomerular filtration rate, MDRD (mL/min/1.73m^2^), mean (SD)	*n = 974*	*n = 748*	*n = 1722*
	61 (42)	81 (29)	70 (38)
Allergy to (any) antibiotics	63 (6)	66 (7)	129 (7)
Treated at outpatient department	30 (3)	620 (63)	650 (33)
Admission at night (7 PM – 7 AM)	*n = 634*	*n = 295*	*n = 929*
	213 (34)	67 (23)	280 (30)
Antibiotic therapy within past 14 days	405 (41)	283 (29)	688 (35)
Febrile UTI	*n = 980*	*n = 982*	*n = 1962*
	794 (81)	386 (39)	1180 (60)
Positive urine culture	*n = 979*	*n = 976*	*n = 1955*
	637 (65)	436 (45)	1073 (55)

^*^Numbers are *n* (%), unless otherwise indicated

**Table 3:** Department characteristics

Departments	Internal Medicine (*n* = 19)	Urology (*n* = 19)	Total (*n* = 38)
Number of beds, mean (SD)	67 (22)	18 (9)	43 (30)
% Female physicians, mean (SD)	33 (13)	17 (15)	25 (16)
Teaching hospital department^*^	17 (89)	12 (63)	29 (76)
Residents working at the department	19 (100)	16 (84)	35 (92)
Microbiological laboratory in the same building as the department	12 (63)	8 (42)	20 (53)
Reporting of a positive urine culture by phone (incidentally or structurally)	0	2 (11)	2 (5)
Structural^a^ education on antibiotics for residents	14 (74)	4 (21)	18 (47)
Structural education on antibiotics for senior staff members	4 (21)	0	4 (11)
Audit and feedback (incidentally^b^ or structurally)			
On antibiotic prescriptions^c^, at the department level	2 (11)	8 (42)	10 (26)
On antibiotic prescriptions^c^, at the individual level	0	2 (11)	2 (5)
On antibiotic resistance rates of the hospital	9 (47)	12 (63)	21 (55)
Individual advice (of microbiologist/ pharmacist/ infectious diseases (ID) physician), incidentally or structurally, regarding streamlining therapy on the basis of blood or urine culture result	16 (84)	16 (84)	32 (84)
Microbiologist and/or ID physician structurally present at ward rounds discussing antibiotic therapy	6 (32)	0	6 (16)
Quality improvement project concerning antibiotic prescribing in past 3 years	8 (42)	7 (37)	15 (40)
Changes in antibiotic procedures or policies in past 3 years^d^	13 (68)	14 (74)	27 (71)

^*^Numbers are *n* (%), unless otherwise indicated

^a^occurring repeatedly at fixed moments in time.

^b^occurring repeatedly but not consequently at fixed moments

^c^feedback on the number and classes of antibiotics prescribed by the individual professional or in the department in a certain time period (not specifically for complicated UTIs)

^d^e.g. culture results became electronically available (while previously on paper), (new) local hospital guideline for complicated UTIs became available, ID physician joined medical staff (while previously no ID physician)

**Table 4:** Hospital characteristics

Hospitals	*n* = 19
Presence of infectious diseases (ID) physician^*^	15 (79)
Teaching hospital for microbiologists	11 (58)
Teaching hospital for ID fellows	4 (21)
Structural ID meetings	13 (68)
Specialism of the chairman of the antibiotic committee	
ID physician	3 (16)
Clinical pharmacologist	2 (11)
Microbiologist	13 (68)
No antibiotic committee	1 (5)
Local resistance rates used in determining local guideline	16 (84)
Antibiotic prescribing policies	
Antibiotic formulary	17 (89)
Restrictive use of certain antibiotics^a^	7 (37)
Selective reporting of culture result	11 (58)
Automatic stop-order	1 (5)
Accessibility of local antibiotic guidelines	
On paper	2 (11)
Digital	9 (47)
Both	7 (37)
None (no local antibiotic guideline)	1 (5)

^*^Numbers are *n* (%), unless otherwise indicated

^a^Permission of a microbiologist or ID physician required before using it

Patient characteristics (Table 2) included 14 variables containing demographic data (age, sex), comorbidities and other characteristics. Comorbidity was defined as having one or more of the following diseases: cardiovascular disease, immunocompromising disease, diabetes mellitus, urological comorbidity, and kidney disease. Urological comorbidity included an anatomical abnormality of the urinary tract (excluding benign prostatic hyperplasia), a history of urolithiasis, or neurological urinary retention.

UTI was classified as ‘febrile UTI’ or ‘non-febrile UTI’. A febrile UTI included pyelonephritis, urosepsis, acute prostatitis and UTI with systemic symptoms (fever, haemodynamic instability or delirium, as described by the attending physician). Non-febrile UTIs were a cystitis/chronic prostatitis in men, UTIs without systemic symptoms in catheterized patients, and cystitis in diabetic or immunocompromised women. A urine culture result was considered ‘positive’ when a bacterial pathogen was regarded as pathogenic (at least 10E4 or 10E5 cfu/ml) by the attending microbiologist and reported together with a susceptibility pattern. A negative culture result and not performing a urine culture were both considered ‘not positive’.

Department characteristics (Table 3) included 15 variables with information regarding general department characteristics, specific routines and already available stewardship elements. Stewardship elements are organizational initiatives aimed at improving quality of antibiotic use [7], e.g. feedback to physicians on antibiotic prescription at the individual or department level.

Hospital characteristics (Table 4) included 11 variables to describe general hospital characteristics and already available stewardship elements at the hospital level, like a restrictive policy for certain antibiotics.

Department characteristics were collected using questionnaires filled in by one internal medicine physician and one urologist of each participating department (June 2010). The internal medicine physician also answered the questionnaire on hospital characteristics.

### Analysis

Not every QI was applicable to all included patients, therefore the sample sizes of the QIs varied. For a QI to be included, we decided that the minimum sample size was a mean of 15 patients per department [21].

Missing values in the possible determinants were imputed using the MICE technique (Multiple Imputation Using Chained Equations). We generated 5 imputed data sets, using the mice package in R [22]. For each QI a unique set of possible determinants was evaluated, because not all determinants were applicable to every QI, e.g. outpatients were never involved in a change from intravenous to oral treatment (see Additional file 1).

Next, the correlation between each pair of determinants was calculated. If two independent variables were highly correlated (correlation coefficient >0.6), only one variable was included in the analysis (see Additional file 1). We also evaluated the consequences of using more rigorous cut off points (correlation coefficient between two determinants >0.4) or less rigorous cut off points (coefficient >0.8).

Multilevel logistic regression analysis was performed, with clusters determined by the unique hospital-department combinations. *P*-values of ≤0.01 were considered statistically significant. Odds ratios were reported to describe associations between determinants and quality indicators (Table 5). For every indicator, we calculated the explained variance: the percentage of variance that the determinants could explain (fit of the models), using a “threshold model formulation” [23].

**Table 5:** Multivariate associations^a^ between patient (P), department (D) and hospital (H) characteristics and QI performance scores, and explained variance.

QI		Odds ratio (95 % CI)	*P*
1	Perform a urine culture		
P	Febrile UTI	1.98 (1.49-2.63)	<0.001
D	Residents working at department	3.38 (1.60-7.14)	0.001
	Explained variance (%)	12.6	
2A	Prescribe empirical therapy according to national guideline		
P	Admission at night	0.61 (0.43-0.86)	0.007
P	Urinary catheter	0.15 (0.10-0.22)	<0.001
H	Presence of infectious diseases (ID) physician	2.52 (1.27-5.01)	0.008
H	Antibiotic formulary	2.98 (1.33-6.67)	0.008
	Explained variance (%)	19.4	
2B	Prescribe empirical therapy according to local guideline		
P	Febrile UTI	1.75 (1.23-2.49)	0.002
P	Antibiotic therapy within past 14 days	0.68 (0.52-0.90)	0.007
P	Urinary catheter	0.47 (0.31-0.71)	<0.001
D	Structural education on antibiotics for senior staff members	10.39 (2.83-38.15)	<0.001
	Explained variance (%)	23.7	
3	Switch from iv to oral therapy within 72 hours on the basis of the clinical condition		
P	Older age	0.85 (0.75-0.95)^b^	0.005
D	Feedback on antibiotic prescription at the department level	0.25 (0.10-0.59)	0.002
D	Higher proportion female physicians	0.70 (0.55-0.90)^c^	0.005
	Explained variance (%)	26.5	
4	Tailor antibiotic treatment on the basis of culture results		
D	Feedback regarding pathogen-directed therapy on the basis of blood or urine culture result	0.23 (0.11-0.48)	<0.001
	Explained variance (%)	11.1	
6A	Duration of antibiotic therapy should be at least 10 days		
P	Febrile UTI	2.22 (1.62-3.05)	<0.001
P	Female patient	0.44 (0.33-0.58)	<0.001
	Explained variance (%)	12.3	
6B	Duration of antibiotic therapy should be according to local guideline		
P	Female patient	0.59 (0.44-0.80)	<0.001
	Explained variance (%)	15.7	
7A	Treat UTI in men according to national guideline		
P	Febrile UTI	2.33 (1.60-3.40)	<0.001
D	Quality improvement project in past 3 years	0.46 (0.27-0.77)	0.004
D	Feedback on antibiotic resistance rates of the hospital	2.07 (1.19-3.61)	0.01
	Explained variance (%)	17.6	
7B	Treat UTI in men according to local guideline		
D	Quality improvement project in past 3 years	0.29 (0.13-0.65)	0.003
	Explained variance (%)	17.1	

^a^Data are presented as Odds Ratios (95 % confidence interval); an Odds Ratio > 1 means a positive association with the QI and an Odds Ratio < 1 means a negative association.

^b^OR per 10 years increase in age (per 1 year increase in age: OR 0.98; 95 % CI: 0.97-1.00).

^c^OR per 10 % increase in proportion female physicians (per % increase: OR 0.97; 95 % CI: 0.94-0.99).

## Results

### Study population

One thousands nine hundred sixty four patients with a complicated UTI were included. The response rate of the questionnaires sent to the participating hospitals, to collect information on department and hospital characteristics, was 100 %. Tables 2, 3 and 4 provide the characteristics of patients, departments and hospitals.

### Quality indicators

**Fig. 1:**
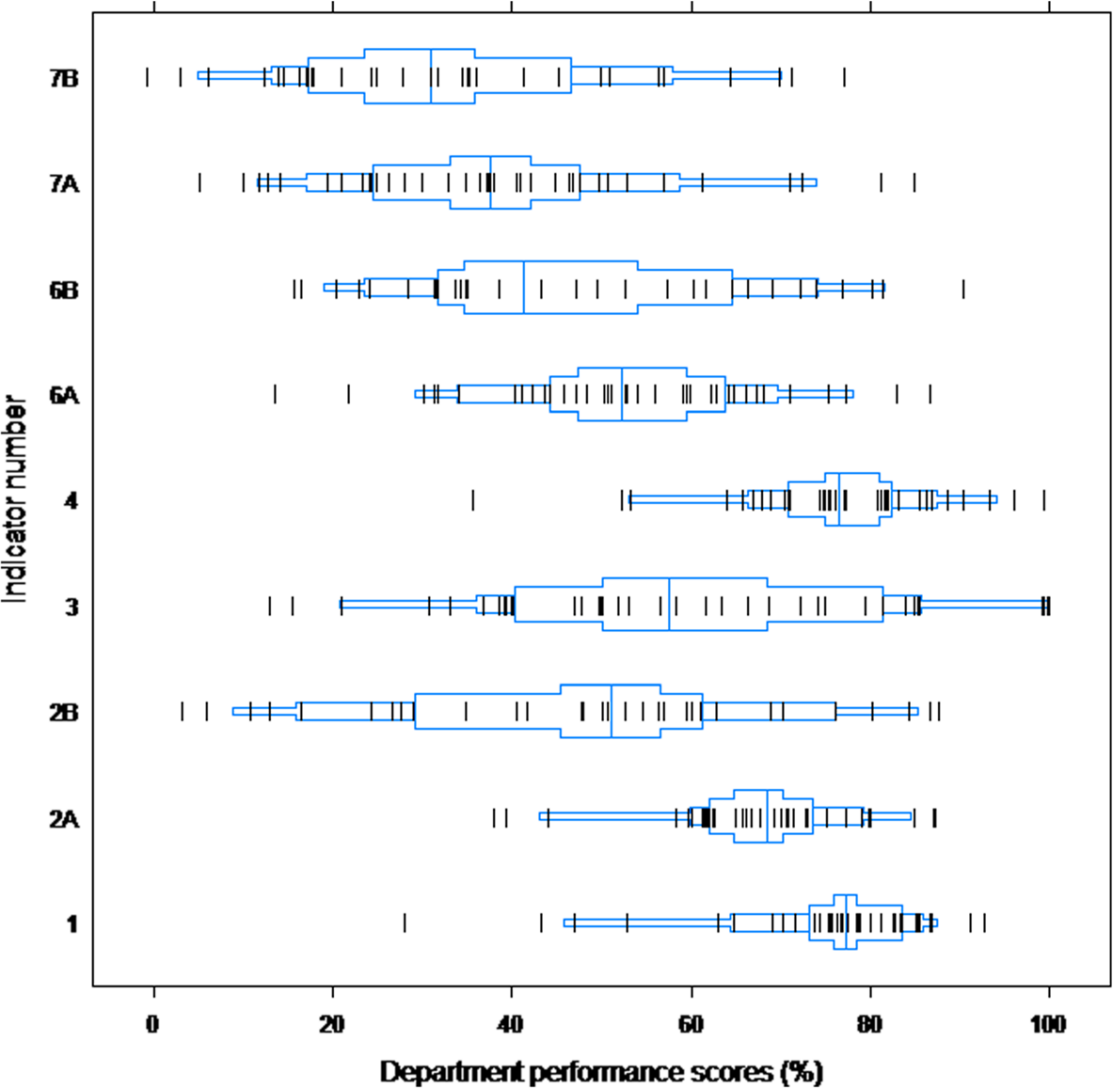
Department performance scores on the QIs. Box-percentiles plots show the proportional distribution. Departments are indicated by vertical black lines ( | ). The box contains 90 percent of all departments. The median and the 30th and 70th percentiles are marked with a vertical blue line

Figure 1 shows the scores of the 38 departments for each of the QIs. Due to the minimum sample size for each QI of a mean of 15 patients per department, three QIs were excluded from the analyses: ‘Use fluoroquinolones selectively’, ‘Replace catheter after initiation of treatment’, and ‘Adapt antibiotic dose according to renal function’. The median QI performance varied between 31 % (‘Treat UTI in men according to local guideline’) and 77 % (‘Perform a urine culture’). Overall, there was a wide inter-department variation. The box-percentiles plots (involving 90 percent of all departments) show that outliers could not explain these wide ranges.

### Determinants of QI scores

Table 5 shows the statistically significant results of multilevel regression analyses to explore determinants for each of the QIs. For also non-significant results see Additional file 2.

#### Perform a urine culture

More urine cultures were performed in patients with a febrile UTI than in patients with a non- febrile UTI. At departments where residents were working, patients also had a greater chance to get their urine cultured.

#### Prescribe empirical therapy in accordance with the guideline

Adherence to the national guideline for complicated UTIs was less common in patients with a urinary catheter and in patients admitted at night. Patients had a better chance to receive national guideline-adherent therapy in hospitals with infectious disease (ID) physicians, or with an antibiotic formulary available.

Subgroups at risk for receiving empirical therapy not according to the local hospital guideline were patients with a urinary catheter, patients who received antibiotic therapy prior to admission and patients with a non-febrile UTI. Adherence to the local hospital guideline was better at departments that organized structural education on antibiotics for senior staff members.

#### Switch from intravenous to oral therapy within 72 hours on the basis of clinical condition

This switch was less likely to be performed in older patients and at departments where more female physicians were working. Providing feedback on antibiotic prescription for all infections at the department level was inversely associated with switching from intravenous to oral therapy within 72 hours.

#### Tailor antibiotic treatment on the basis of culture results

At departments where internal medicine physicians or urologists received feedback about pathogen-directed therapy after determination of the blood or urine culture results, patients received less culture-guided, tailored therapy.

#### Duration of antibiotic therapy should be in accordance with the guideline

Patients with a febrile UTI were more likely to receive antibiotics for the duration recommended in the national guideline. Treatment duration was less appropriate in female patients: in general their treatment durations were shorter than recommended in the national (10 days) and local hospital guidelines (varying between 7 -14 days).

#### Treat UTI in men in accordance with the guideline

Men with a febrile UTI were more likely to be treated in accordance with the guideline than men with a non-febrile UTI (definition of this QI see: Method section, *Variables and data collection*). Providing feedback on antibiotic resistance rates of the hospital was positively associated with adherence to the national guideline. However, quality improvement projects in the past three years concerning antibiotic prescribing were inversely associated with this QI. This negative association was also shown for the QI ‘Treat every man in accordance with the local guideline’.

Using more (correlation coefficient > 0.4) or less (correlation coefficient > 0.8) rigorous cut off points for the maximal correlation between every pair of determinants hardly changed our results.

The explained variance for each indicator is also shown in Table 5. Variation between departments in QI performance scores regarding the QIs ‘Prescribe empirical therapy in accordance with the (national/local hospital) guideline’ and ‘Switch from intravenous to oral therapy within 72 hours’ could be explained in part by the evaluated patient, department and hospital characteristics, with explained variances of 19, 24 and 27 %, respectively.

## Discussion

In this study, we systematically assessed appropriateness of antibiotic use in patients with a complicated UTI, using a set of validated quality indicators. A large variation was demonstrated between departments in performance scores of the QIs. Various determinants, in particular patient characteristics, influenced the quality of antibiotic use.

The relatively poor rates of guideline adherence and the large inter-department variation found in the present study confirm data from earlier reports in patients with pneumonia and uncomplicated UTIs [20–24]. The variation in QI performance scores could only be partly explained by the evaluated patient, department and hospital characteristics, with explained variances ranging from 11 to 27 %. These figures are comparable with those found in other studies exploring determinants of appropriate care [18–20].

As far as we know, this is the first study in which the entire process of the antibiotic treatment of complicated UTI is studied. In previous studies, factors influencing antibiotic use have been evaluated, but complicated UTIs were not investigated, and most of them focused on a single aspect of the antibiotic treatment.

In particular certain patient characteristics influenced the quality of antibiotic use. Our finding that urine cultures were more likely to be performed in patients with a febrile UTI are in line with studies in patients with pneumonia, in which parameters that reflect severity of disease were positively associated with the collection of blood cultures [16, 20, 25]. In our study, patients with a febrile UTI had also better chances to receive the right antibiotic agent and treatment duration, and men with a febrile UTI were more likely to be treated in accordance with the guideline. An explanation for the more appropriate treatment of patients with a febrile UTI could be that they are a rather uniform group, and as such more easily recognized as having a complicated UTI. In contrast, patients with a non-febrile UTI are a more diverse group: male patients, and patients with an abnormality of the urinary tract or with an immunodeficiency, who can be mistakenly regarded as having an uncomplicated UTI. Furthermore, in international guidelines regarding the diagnosis and treatment of men with cystitis/prostatitis and patients with urinary catheter-associated UTIs, high-quality evidence is lacking and recommendations are often based on expert opinion [26, 27].

There was a strong negative association between having a urinary catheter and prescribing empirical therapy in accordance with both the national and local hospital guidelines, which include specific treatment recommendations for these patients. Other patient characteristics associated with inappropriate antibiotic use were age (older age: less switching) and gender (females: less appropriate treatment duration). An explanation for the negative association of female gender of the patient with receiving the recommended treatment duration could be that some studies suggested shorter treatment to be appropriate in premenopausal, nonpregnant women with acute pyelonephritis [28, 29].

Concerning department and hospital characteristics, including available stewardship elements, the presence of an ID physician was positively associated with prescribing empirical therapy in accordance with the national guideline. This is in line with the growing evidence that ID physicians play an important role in patient care, infection control and antibiotic management [30]. Other stewardship elements associated with more appropriate antibiotic use were the presence of an antibiotic formulary, feedback on antibiotic resistance rates of the hospital and structural education on antibiotics for senior staff members. Surprisingly, a few stewardship elements were inversely associated with appropriate antibiotic use, e.g. feedback about pathogen-directed therapy was associated with less culture-guided antibiotic therapy. A possible explanation for this paradox is that we asked for feedback on culture results in general, not specifically for feedback on UTIs. Hypothetically, if feedback on blood cultures was structurally provided, but feedback on urine cultures not (or incidentally), it might have had an inverse effect on tailoring therapy for UTIs. The association between quality improvement projects in the past three years and less appropriate treatment of UTI in men is another example of such a paradox. It is important to realize that we asked for projects concerning antibiotic prescribing, not specifically for UTIs or UTIs in men, so unintended effects (concerning this QI) might have played a role. Overall, no hospital or departments characteristics, including stewardship elements, were consistently associated with better performance on the QIs. With the implementation of antibiotic stewardship programs at a large scale, our study underlines the need of careful evaluation of the effects of different clearly described stewardship elements, to assess their effect in daily clinical practice. However, we should be aware that antibiotic stewardship programs comprise the introduction of a menu of different interventions [7], whose effect could be more than the sum of effects of single (available) stewardship elements. In addition, we measured QI performance scores, not clinical endpoints.

The strength of the study is the rigorous and objective assessment of appropriate antibiotic use, using a systematically developed set of guideline-based QIs, describing the entire process of antibiotic use in complicated UTIs, from admission to discharge. We earlier demonstrated that better performance on the total set of QIs was associated with a shorter length of hospital stay [31]. The large sample of 1964 patients from 19 different hospitals all over the Netherlands contributes to the validity of the results. Care for UTI patients is performed at the in- and outpatient departments of internal medicine and urology. Since patients from both departments were included, our findings are largely representative for the entire UTI population. The study is based on the accurate diagnosis by treating physicians who can be notoriously inaccurate in diagnosing UTIs [32], however, the study demonstrates real life application of the UTI guideline.

Our study has limitations. First, department and hospital characteristics were collected from questionnaires sent to an internist and a urologist of each participating hospital. This may have introduced bias, through issues of social desirability. However, the majority of the questions concerned basic factual information. Second, characteristics of the individual treating physician, which could also contribute to variation in appropriate antibiotic use (e.g. clinical experience, membership of local antibiotic committee) were not measured, since most patients during admission were treated by a dynamic team of senior staff members and residents. A final limitation of the study was the explorative design used to identify possible determinants. Owing to this design, associations can be demonstrated, but causal relationships cannot be inferred.

## Conclusion

A large inter-department variation was demonstrated in the appropriateness of antibiotic use. In particular patient characteristics were associated with the risk to receive less appropriate antibiotic treatment. Some, but not all antibiotic stewardship elements did translate into better QI performance.

## Additional files

Additional file 1:
**Characteristics included in multivariable model per QI.** (DOC 73 kb) Additional file 2:
**Multivariate analysis of patient, department and hospital characteristics and QI performance scores (significant and non-significant results).** (DOCX 40 kb)

## Abbreviations

QI: Quality indicator
UTI: Urinary tract infection
ID physician: Infectious diseases physician
