# Supplementary material for: Appropriate antibiotic use for patients with complicated urinary tract infections in 38 Dutch Hospital Departments: a retrospective study of variation and determinants
Source: BMC Infect Dis. 2015 Nov 9;15:505. doi: 10.1186/s12879-015-1257-5 (PMC4640398; doi:10.1186/s12879-015-1257-5)
Supplement: Additional file 1: — Characteristics included in multivariable model per QI. (DOC 73 kb) [file 12879_2015_1257_MOESM1_ESM.doc]

**Additional File 1**

Table 1. Characteristics included (+) in multivariable model per QI. In bold the characteristics that were studied for all QIs.

| Quality Indicator (number)* | 1 | 2 | 3 | 4 | 6 | 7 |
| --- | --- | --- | --- | --- | --- | --- |
|  |  |  |  |  |  |  |
| **Age** | + | + | + | + | + | + |
| Sex | + | + | + | + | + | na^a^ |
| **Comorbidity** | + | + | + | + | + | + |
| **Urological comorbidity** | + | + | + | + | + | + |
| **Diabetes** | + | + | + | + | + | + |
| Urinary catheter | + | + | + | + | + | na |
| **Mean glomerular filtration rate, MDRD** | + | + | + | + | + | + |
| **Allergy to (any) antibiotics** | + | + | + | + | + | + |
| Outpatient/inpatient | C^b^ | C^b^ | na | C^b^ | C^b^ | C^b^ |
| **Admission at night (7 PM – 7 AM)** | + | + | + | + | + | + |
| **Antibiotic therapy within past 14 days** | + | + | + | + | + | + |
| **Primary diagnosis (febrile UTI/ non-febrile UTI )** | + | + | + | + | + | + |
| Urine culture (positive/negative) | na | na | + | na | + | + |
| **Department (internal medicine/ urology)** | + | + | + | + | + | + |
| Mean number of beds | C^c^ | C^c^ | C^c^ | C^c^ | C^c^ | C^c^ |
| **Mean % female physicians** | + | + | + | + | + | + |
| **Teaching hospital department** | + | + | + | + | + | + |
| **Residents working at department** | + | + | + | + | + | + |
| Microbiological laboratory in the same building as the department | C^d^ | na | C^d^ | C^d^ | na | C^d^ |
| Reporting of a positive urine culture by phone | + | na | + | + | na | na |
| **Structural education on antibiotics for residents** | + | + | + | + | + | + |
| **Structural education on antibiotics for senior staff members** | + | + | + | + | + | + |
| Feedback on antibiotic prescription at department level | na | + | + | + | + | + |
| Feedback on antibiotic prescription at individual level | na | + | + | + | + | + |
| Feedback on antibiotic resistance rates of the hospital | na | + | + | + | + | + |
| Individual advice regarding streamlining therapy | na | na | + | + | + | + |
| **Microbiologist and/or infectious diseases (ID) physician structurally present at ward rounds discussing antibiotics** | + | + | + | + | + | + |
| **Quality improvement project concerning antibiotic prescribing in past 3 years** | + | + | + | + | + | + |
| **Changes in antibiotic procedures or policies in past 3 years** | + | + | + | + | + | + |
| **Presence of ID physician** | + | + | + | + | + | + |
| Teaching hospital for microbiologists | + | C^e^ | C^e^ | C^e^ | C^e^ | C^e^ |
| **Teaching hospital for ID fellows** | + | + | + | + | + | + |
| **Structural ID meetings** | + | + | + | + | + | + |
| Specialism of the chairman of the antibiotic committee (ID physician, clinical pharmacologist, microbiologist) | + | na | + | + | na | C^f^ |
| Local resistance rates used in determining local guideline | na | + | na | na | na | + |
| Antibiotic formulary | na | + | + | + | na | + |
| Restrictive use of certain antibiotics | na | + | + | + | na | + |
| Selective reporting of culture result | na | na | + | + | na | + |
| Automatic stop-order | na | na | na | na | + | + |
| Accessibility of local antibiotic guidelines (on paper, digital, both) | na | + | na | na | + | na |
| Total number of studied determinants | 26 | 30 | 33 | 32 | 30 | 31 |

* see Box 1 for explanation of the QI numbers

^a^ na = the determinant was not applicable (na) to the indicated QI(s)

^b^C = the determinant ‘Outpatient/inpatient’ was highly Correlated (i.e. correlation

coefficient >0.6) with other determinants (i.e.: ‘Primary diagnosis’ and ‘Department’)

and excluded for QIs 1, 2, 4, 6 and 7

^c^C = the determinant ‘Mean number of beds’ was highly Correlated with another determinant

(i.e.: ‘Department’) and excluded for all QIs

^d^C = the determinant ‘Microbiological laboratory’ was highly Correlated with other

determinants (i.e.: ‘Structural ID meetings’ and ‘Restrictive use of antibiotics’) and

excluded for QIs 1, 3, 4 and 7

^e^C = the determinant ‘Teaching hospital microbiologists’ was highly Correlated with other

determinants (i.e.: ‘Selective reporting’ and ‘Accessibility of local guidelines’) and excluded for QIs 2, 3, 4, 6 and 7

^f^C = the determinant ‘Specialism of the chairman’ was highly Correlated with other

determinants (i.e.: ‘Local resistance rates used in local guideline’ and

‘Automatic stop-order’) and excluded for QI 7
