# Supplementary material for: Appropriate antibiotic use for patients with complicated urinary tract infections in 38 Dutch Hospital Departments: a retrospective study of variation and determinants
Source: BMC Infect Dis. 2015 Nov 9;15:505. doi: 10.1186/s12879-015-1257-5 (PMC4640398; doi:10.1186/s12879-015-1257-5)
Supplement: Additional file 2: — Multivariate analysis of patient, department and hospital characteristics and QI performance scores (significant and non-significant results). (DOCX 40 kb) [file 12879_2015_1257_MOESM2_ESM.docx]

**Additional File 2**

Tables 1-9: Multivariate analysis of patient, department and hospital characteristics and QI performance scores (in bold the statistically significant characteristics, P-values ≤ 0.01).

| QI 1: Perform a urine culture |
| --- |
| Odds P 95% CI  Department Urology 0.6102622 6.118595e-02 0.3638832 1.0234604  Age 1.0024840 4.854217e-01 0.9955126 1.0095041  Admission at night 0.7034810 3.607186e-02 0.5069550 0.9761922  Comorbidity 1.5331403 1.259467e-02 1.0960856 2.1444667  Urological comorbidity 0.9470357 7.592403e-01 0.6685380 1.3415491  Diabetes 0.8664903 4.087814e-01 0.6166413 1.2175724  Allergy to (any) antibiotics 0.9977619 9.921734e-01 0.6375224 1.5615587  Antibiotic therapy < 14 days 1.1548004 2.459304e-01 0.9054798 1.4727706  **Febrile UTI 1.9786211 2.806451e-06 1.4885074 2.6301121**  Teaching hospital department 0.8730316 6.282470e-01 0.5037451 1.5130353  Mean % female physicians 0.9897633 1.057430e-01 0.9774985 1.0021820  Microbiologist and/or ID  physician present at ward rounds  discussing antibiotics 0.6990561 1.424118e-01 0.4332210 1.1280140  **Residents working at department 3.3794093 1.453651e-03 1.5987101 7.1435133**  Structural education on  antibiotics for residents 0.7111203 1.774286e-01 0.4332304 1.1672588  Structural education on  antibiotics for senior staff  members 1.0404370 8.913478e-01 0.5889360 1.8380757  Quality improvement project  concerning antibiotics < 3yrs 1.1807589 3.463540e-01 0.8354305 1.6688301  Changes in antibiotic procedures  or policies < 3yrs 1.0105529 9.582459e-01 0.6820166 1.4973493  Presence of ID physician 0.7774957 3.351538e-01 0.4659438 1.2973658  Teaching hospital microbiologists 1.0433588 8.700978e-01 0.6271816 1.7356976  Teaching hospital ID fellow 1.6522459 1.868618e-02 1.0873497 2.5106151  Structural ID meetings 1.1605393 5.208137e-01 0.7364496 1.8288440  Female 0.9687222 8.296295e-01 0.7251531 1.2941029  Urinary catheter 1.2588789 2.350841e-01 0.8607647 1.8411258  Mean Glomerular filtration rate 0.9997139 8.829549e-01 0.9958257 1.0036173  Chairman of antibiotic committee  ID physician 0.5095245 4.631279e-02 0.2624966 0.9890232  Clinical pharmacologist 0.6152997 6.864551e-02 0.3647796 1.0378699  Microbiologist 0.3225229 4.139141e-02 0.1087244 0.9567406  Reporting positive urine culture  by phone 1.1835191 6.589438e-01 0.5593963 2.5039804 |

| QI 2A: Prescribe empirical therapy according to national guideline |
| --- |
| Odds P 95% CI  Department Urology 0.9133521 0.704382445 0.5716965 1.4591868  Age 1.0011846 0.751776861 0.9938602 1.0085631  **Admission at night 0.6088303 0.006993342 0.4293874 0.8632630**  Comorbidity 0.7460261 0.097218794 0.5276501 1.0547802  Urological comorbidity 1.2395628 0.231737035 0.8716281 1.7628114  Diabetes 1.0719884 0.690015263 0.7616052 1.5088647  Allergy to (any) antibiotics 0.6582840 0.092525212 0.4043963 1.0715671  Antibiotic therapy < 14 days 0.8331167 0.153797933 0.6481887 1.0708046  Febrile UTI 1.3685969 0.052807096 0.9961983 1.8802056  Teaching hospital department 0.3809805 0.042992234 0.1496387 0.9699774  Mean % female physicians 0.9888877 0.077588373 0.9766899 1.0012378  Microbiologist and/or ID  physician present at ward rounds  discussing antibiotics 0.8148209 0.516331534 0.4388448 1.5129108  Residents working at department 1.7310481 0.399821489 0.4812564 6.2264683  Structural education on  antibiotics for residents 1.0457147 0.867436986 0.6184368 1.7681987  Structural education on  antibiotics for senior staff  members 0.9853804 0.963787475 0.5215045 1.8618719  Quality improvement project  concerning antibiotics < 3yrs 0.7777089 0.155813261 0.5494931 1.1007074  Changes in antibiotic procedures  or policies < 3yrs 0.8506858 0.452907721 0.5573926 1.2983062  **Presence of ID physician 2.5240731 0.008081187 1.2727071 5.0058220**  Teaching hospital ID fellows 1.7175077 0.034816774 1.0393936 2.8380323  Structural ID meetings 0.7553510 0.281760542 0.4530160 1.2594589  Female 1.0586819 0.702055243 0.7902960 1.4182120  **Urinary catheter 0.1475243 0.000000000 0.1011597 0.2151393**  Mean Glomerular filtration rate 0.9998152 0.920035257 0.9962034 1.0034401  Feedback on antibiotic  prescription at department level 0.9589407 0.847518649 0.6252687 1.4706753  Feedback on antibiotic  prescription at individual level 1.0340709 0.935738408 0.4576634 2.3364392  Feedback on antibiotic  resistance rates of the hospital 0.9494265 0.814596622 0.6150449 1.4656014  Local resistance rates used in  determining local guideline 1.9641065 0.034294185 1.0513054 3.6694516  Accessibility of local guidelines  On paper 0.4602951 0.024047766 0.2346436 0.9029508  Digital 1.0038540 0.985177174 0.6687768 1.5068149  Both 0.8842585 0.829337901 0.2887240 2.7081685  **Antibiotic formulary 2.9800307 0.008118268 1.3304750 6.6747464**  Restrictive use of antibiotics 0.7250071 0.264409764 0.4120544 1.2756455 |

| QI 2B: Prescribe empirical therapy according to local guideline |
| --- |
| Odds P 95% CI  Department Urology 0.68182548 0.4394794658 0.258044368 1.8015738  Age 1.00125282 0.7568652240 0.993341796 1.0092268  Admission at night 0.89628559 0.5700754464 0.607231416 1.3229353  Comorbidity 0.76417071 0.1601437278 0.524963153 1.1123769  Urological comorbidity 0.88912885 0.5436001475 0.608352753 1.2994929  Diabetes 1.00484546 0.9799130556 0.689521686 1.4643693  Allergy to (any) antibiotic 0.54155651 0.0278525399 0.313566755 0.9353143  **Antibiotic therapy < 14 days 0.68330831 0.0065595890 0.519359718 0.8990113**  **Febrile UTI 1.75267146 0.0017488984 1.233931598 2.4894875**  Teaching hospital department 3.65956533 0.1262237308 0.693485931 19.3117377  Mean % female physicians 0.98506599 0.2729078538 0.958908206 1.0119373  Microbiologist and/or ID  physician present at ward  rounds discussing antibiotics 2.99221500 0.1266681885 0.732807271 12.2178790  Residents working at  department 0.03296954 0.0163341748 0.002037644 0.5334548  Structural education on  antibiotics for residents 0.31985345 0.0512974478 0.101650220 1.0064536  **Structural education on**  **antibiotics for senior staff**  **members 10.39424122 0.0004273572 2.831940167 38.1506120**  Quality improvement project  concerning antibiotics < 3yrs 0.37978613 0.0213889423 0.166538362 0.8660917  Changes in antibiotic  Procedures or policies < 3yrs 0.64211939 0.2857706290 0.284557558 1.4489769    Presence of ID physician 0.43378729 0.1686625515 0.132006361 1.4254723  Teaching hospital ID fellows 2.90094809 0.0226585660 1.161155667 7.2475208  Structural ID meetings 1.22599834 0.7873019367 0.278724174 5.3926859  Female 0.88732759 0.4582019034 0.646871196 1.2171670  **Urinary catheter 0.47229017 0.0003383167 0.313609450 0.7112605**  Mean Glomerular filtration 1.00174508 0.3564944047 0.998036898 1.0054670  Feedback on antibiotic  prescription at department  level 1.19233530 0.7303331456 0.438157172 3.2446427  Feedback on antibiotic  prescription at individual  level 3.41438632 0.1278479377 0.702430073 16.5967182  Feedback on antibiotic  resistance rates of hospital 2.42732970 0.0685935285 0.934509143 6.3048388  Local resistance rates used  in determining local  guideline 0.15103529 0.0193867287 0.030983851 0.7362435  Accessibility local guidelines  On paper 0.32694697 0.1204865296 0.079695102 1.3412910  Both (paper and digital) 1.85184839 0.1291278670 0.835352739 4.1052627  Restrictive use antibiotics 5.64181981 0.0150910268 1.398497136 22.7602402  Antibiotic formulary 0.37351243 0.2284994349 0.075135724 1.8567937 |

| QI 3: Switch from iv to oral therapy within 72 hours on the basis of clinical condition |
| --- |
| Odds P 95% CI  Department Urology 1.1018176 0.842133124 0.423539850 2.8663229  **Age 0.9834457 0.005204549 0.972021077 0.9950047**  Admission at night 1.4504001 0.113658614 0.914127404 2.3012772  Comorbidity 1.6140779 0.086451437 0.933487489 2.7908755  Urological comorbidity 0.6746977 0.143842781 0.397879783 1.1441068  Diabetes 0.7181448 0.201688389 0.431745125 1.1945287  Allergy to (any) antibiotic 0.3624731 0.036842668 0.139826348 0.9396423  Antibiotic therapy < 14 days 0.9007024 0.609091911 0.602799134 1.3458296  Febrile UTI 0.7397313 0.365494984 0.384649145 1.4226013  Teaching hospital department 0.2726725 0.225438510 0.033267793 2.2349038  **Mean % female physicians 0.9655924 0.004718947 0.942472926 0.9892790**  Microbiologist and/or ID  physician present at ward  rounds discussing antibiotics 1.1916874 0.722970989 0.451082203 3.1482483  Residents working at  department 1.6142926 0.772077580 0.062797788 41.4973286  Structural education on  antibiotics for residents 0.6197625 0.269404671 0.264848706 1.4502827  Structural education on  antibiotics for senior staff  members 2.4185694 0.051865919 0.992856414 5.8915647  Quality improvement project  concerning antibiotics < 3yrs 2.3561016 0.014677322 1.184632019 4.6860245  Changes in antibiotic procedures  or policies < 3yrs 2.1296290 0.075699540 0.924494246 4.9057306  Presence of ID physician 1.2746426 0.683785455 0.395621371 4.1067392  Teaching hospital ID fellows 0.7807349 0.539351564 0.353693093 1.7233783  Structural ID meetings 2.0003840 0.257209976 0.601871996 6.6484840    Female 0.9586289 0.852305277 0.613901327 1.4969334  Urinary catheter 1.1521836 0.634944636 0.641319032 2.0699947  Mean Glomerular filtration rate 1.0058130 0.098175743 0.998923125 1.0127503  Urine culture positive 0.5870982 0.033376005 0.359512566 0.9587545  **Feedback on antibiotic**  **prescription at department**  **level 0.2450539 0.001685910 0.102165922 0.5877832**  Feedback on antibiotic  prescription at individual  level 1.8121814 0.533408130 0.278098907 11.8087540  Feedback on antibiotic  resistance rates of hospital 0.4938939 0.121802973 0.201955887 1.2078441  Reporting positive urine culture  by phone 0.3815220 0.355073771 0.049331670 2.9506210  Individual advice regarding  streamlining therapy 0.5847702 0.265982403 0.226914968 1.5069795  Antibiotic formulary 4.1677535 0.226334128 0.411553402 42.2063559  Restrictive use antibiotics 0.2605619 0.046242528 0.069437280 0.9777529  Selective reporting of culture  result 1.0212049 0.964027794 0.409536471 2.5464385  Chairman of antibiotic committee  ID physician 0.1789300 0.245629378 0.009760604 3.2801187 Clinical pharmacologist 0.3052911 0.056402966 0.090222278 1.0330336  Microbiologist 0.7066322 0.751343452 0.082145635 6.0785828 |
| QI 4: Tailor antibiotic treatment on the basis of culture results |
| Odds P 95% CI  Department Urology 0.7001238 2.589102e-01 0.3768982 1.3005456  Age 0.9916422 6.455467e-02 0.9828550 1.0005080  Admission at night 0.7625525 1.297970e-01 0.5364528 1.0839469  Comorbidity 1.6110460 2.294379e-02 1.0682797 2.4295782  Urological comorbidity 0.6115776 1.343976e-02 0.4142103 0.9029887  Diabetes 0.8400753 3.702422e-01 0.5736609 1.2302156  Allergy to (any) antibiotic 1.0781579 7.945406e-01 0.6116380 1.9005106  Antibiotic therapy < 14 days 1.1719885 3.084565e-01 0.8633738 1.5909181  Febrile UTI 0.7586536 1.330973e-01 0.5289951 1.0880162  Teaching hospital department 0.7950264 7.257810e-01 0.2204062 2.8677361  Mean % female physicians 0.9867243 7.639652e-02 0.9722431 1.0014211  Microbiologist and/or ID  physician present at ward  rounds discussing antibiotics 1.5732362 1.963141e-01 0.7909276 3.1293286  Residents working at department 1.4023152 7.177506e-01 0.2238285 8.7856890  Structural education on  antibiotics for residents 0.6529753 1.505422e-01 0.3651179 1.1677781  Structural education on  antibiotics for senior staff  members 1.1913708 6.395342e-01 0.5721518 2.4807477  Quality improvement project  concerning antibiotics < 3yrs 0.9388597 7.976451e-01 0.5793845 1.5213689  Changes in antibiotic procedures  or policies < 3yrs 0.6535609 1.201818e-01 0.3821573 1.1177121  Presence of ID physician 1.2466047 5.184850e-01 0.6381305 2.4352753  Teaching hospital ID fellows 1.3711808 2.613460e-01 0.7901798 2.3793785  Structural ID meetings 1.3416984 4.792022e-01 0.5940009 3.0305584    Female 0.7900702 1.664220e-01 0.5657934 1.1032488  Urinary catheter 0.8137689 2.996164e-01 0.5511581 1.2015061  Mean Glomerular filtration rate 1.0000277 9.918887e-01 0.9946820 1.0054021  Feedback on antibiotic  prescription at department  level 1.2269243 4.330348e-01 0.7355546 2.0465419  Feedback on antibiotic  prescription at individual  level 1.1197102 8.078466e-01 0.4498023 2.7873378  Feedback on antibiotic  resistance rates of hospital 1.3316468 3.882679e-01 0.6943945 2.5537113  Reporting positive urine culture  by phone 1.4386723 5.961626e-01 0.3742789 5.5300421  **Individual advice regarding**  **streamlining therapy 0.2328434 6.882728e-05 0.1138309 0.4762856**  Chairman of antibiotic committee  ID physician 2.3619827 3.779856e-01 0.3490084 15.9851802  Clinical pharmacologist 0.7123056 4.363478e-01 0.3029699 1.6746858  Microbiologist 0.6871702 5.892717e-01 0.1758217 2.6856919  Antibiotic formulary 1.6850358 4.981321e-01 0.3719183 7.6343254  Restrictive use antibiotics 1.0867973 8.535398e-01 0.4487632 2.6319634  Selective reporting of culture  result 1.4486670 2.264900e-01 0.7942721 2.6422128 |

| QI 6A: Duration of antibiotic therapy should be at least 10 days |
| --- |
| Odds P 95% CI  Department Urology 0.8262479 4.927857e-01 0.4787533 1.4259654  Age 0.9933310 5.048403e-02 0.9866925 1.0000141  Admission at night 0.8731767 3.989238e-01 0.6314019 1.2075313  Comorbidity 0.7683054 9.793274e-02 0.5622723 1.0498352  Urological comorbidity 1.0190185 9.053351e-01 0.7468701 1.3903337  Diabetes 1.0280630 8.615865e-01 0.7530368 1.4035351  Allergy to (any) antibiotic 0.9841185 9.421132e-01 0.6386512 1.5164602  Antibiotic therapy < 14 days 0.8741156 2.532346e-01 0.6938852 1.1011592  **Febrile UTI 2.2236145 7.920840e-07 1.6209421 3.0503626**  Teaching hospital department 1.8087148 8.892843e-02 0.9136574 3.5806080  Mean % female physicians 0.9962049 6.225278e-01 0.9812279 1.0114104  Microbiologist and/or ID  physician present at ward  rounds discussing antibiotics 1.3160129 3.792955e-01 0.7132783 2.4280706  Residents working at department 2.4148513 1.053633e-01 0.8305654 7.0211289  Structural education on  antibiotics for residents 1.0808749 7.613937e-01 0.6540757 1.7861704  Structural education on  antibiotics for senior staff  members 0.9208013 7.933893e-01 0.4964061 1.7080271  Quality improvement project  concerning antibiotics < 3yrs 0.7736193 2.099729e-01 0.5178230 1.1557748  Changes in antibiotic procedures  or policies < 3yrs 0.6583951 6.597599e-02 0.4216721 1.0280122  Presence of ID physician 1.1586275 6.486372e-01 0.6147885 2.1835439  Teaching hospital ID fellows 1.3468836 2.751570e-01 0.7887560 2.2999451  Structural ID meetings 1.2360344 3.648245e-01 0.7814039 1.9551747  **Female 0.4379620 5.818848e-09 0.3321603 0.5774642**  Urinary catheter 1.0788179 6.969574e-01 0.7362334 1.5808141  Mean Glomerular filtration rate 0.9949816 1.589196e-02 0.9909503 0.9990293  Urine culture positive 1.0266159 8.202225e-01 0.8183850 1.2878295  Feedback on antibiotic  prescription at department  level 0.9171011 7.160691e-01 0.5751324 1.4624013  Feedback on antibiotic  prescription at individual  level 0.4759506 5.613985e-02 0.2221617 1.0196581  Feedback on antibiotic  resistance rates of hospital 1.1182755 6.228132e-01 0.7161036 1.7463115  Individual advice regarding  streamlining therapy 0.7836668 3.990608e-01 0.4445749 1.3813953  Accessibility of local guidelines  On paper 1.0534242 8.991076e-01 0.4709386 2.3563637  Digital 0.7395018 1.621816e-01 0.4842910 1.1292032  Both 1.1893643 7.642993e-01 0.3825513 3.6977724  Automatic stop-order 3.1577142 1.406706e-02 1.2616489 7.9032755 |

| QI 6B: Duration of antibiotic therapy should be according to local guideline |
| --- |
| Odds P 95% CI  Department Urology 1.0823650 0.8552828311 0.46205819 2.5354252  Age 0.9957677 0.2722321892 0.98825254 1.0033400  Admission at night 0.8587347 0.3883266810 0.60361861 1.2216741  Comorbidity 0.8255340 0.2818310185 0.58212261 1.1707264  Urological comorbidity 1.0898222 0.6287654498 0.76878872 1.5449139  Diabetes 1.1371967 0.4716860264 0.80109765 1.6143054  Allergy to (any) antibiotic 1.0911368 0.7215565305 0.67515252 1.7634230  Antibiotic therapy < 14 days 1.1853827 0.2009061174 0.91331941 1.5384892  Febrile UTI 0.6898914 0.0368203982 0.48690588 0.9774994  Teaching hospital department 1.3187660 0.5979965311 0.47116667 3.6911433  Mean % female physicians 0.9949352 0.6731604816 0.97171948 1.0187057  Microbiologist and/or ID  physician present at ward  rounds discussing antibiotics 1.4991627 0.4201542522 0.55981370 4.0147087  Residents working at department 0.9203368 0.9404400195 0.10409858 8.1367081  Structural education on  antibiotics for residents 1.4501996 0.3504254289 0.66433925 3.1656699  Structural education on  antibiotics for senior staff  members 1.0800302 0.8766905105 0.40810501 2.8582478  Quality improvement project  concerning antibiotics < 3yrs 0.7849759 0.4791057718 0.40131514 1.5354199  Changes in antibiotic  procedures or policies < 3yrs 1.3545548 0.3782756342 0.68934045 2.6617017  Presence of ID physician 2.7407522 0.0387596015 1.05355155 7.1299049  Teaching hospital ID fellows 1.8530221 0.1369635308 0.82172720 4.1786264  Structural ID meetings 1.0483189 0.9002774365 0.50085493 2.1941932  **Female 0.5902270 0.0005533631 0.43780044 0.7957231**  Urinary catheter 0.8985635 0.6135428958 0.59312284 1.3612970  Mean Glomerular filtration 0.9962339 0.0843513669 0.99197279 1.0005134  Urine culture positive 1.3634892 0.0185850354 1.05333303 1.7649716  Feedback on antibiotic  prescription at department  level 1.2795222 0.5427094562 0.57827643 2.8311321  Feedback on antibiotic  prescription at individual  level 0.3464795 0.0741167749 0.10824834 1.1090060  Feedback on antibiotic  resistance rates of hospital 0.3911203 0.0145213323 0.18429635 0.8300495  Individual advice regarding  streamlining therapy 1.2151693 0.6700448420 0.49548384 2.9801908  Accessibility of local guidelines  On paper 0.7494520 0.6492333876 0.21606906 2.5995311  Both (paper and digital) 0.7430664 0.3846882737 0.38024011 1.4521027    Automatic stop-order 1.0131355 0.9852628807 0.25340894 4.0505416 |

| QI 7A: Treat UTI in men according to national guideline |
| --- |
| Odds P 95% CI  Department Urology 0.5787851 8.350360e-02 0.31153331 1.0753013  Age 0.9964951 5.318161e-01 0.98556503 1.0075464  Admission at night 0.8752207 7.095077e-01 0.39678247 1.9305571  Comorbidity 0.8589500 5.020272e-01 0.55078719 1.3395285    Urological comorbidity 1.1051173 6.692991e-01 0.69821076 1.7491626  Diabetes 0.9482341 8.336641e-01 0.57706608 1.5581368  Allergy to (any) antibiotic 1.3266628 4.765484e-01 0.60874098 2.8912692  Antibiotic therapy < 14 days 0.8860025 4.941195e-01 0.62605241 1.2538892  **Febrile UTI 2.3340972 1.187639e-05 1.60025140 3.4044711**  Teaching hospital department 1.7375631 2.479768e-01 0.68009134 4.4392942  Mean % female physicians 0.9898268 1.570977e-01 0.97589786 1.0039546  Microbiologist and/or ID  physician present at ward  rounds discussing antibiotics 1.1670220 6.929242e-01 0.54172628 2.5140745  Residents working at department 1.6067121 5.444920e-01 0.34604591 7.4600611  Structural education on  antibiotics for residents 0.7823055 5.023124e-01 0.38154736 1.6039997  Structural education on  antibiotics for senior staff  members 1.8922824 1.936063e-01 0.72295685 4.9528994  **Quality improvement project**  **concerning antibiotics < 3yrs 0.4582204 3.544134e-03 0.27148335 0.7734026**  Changes in antibiotic  procedures or policies < 3yrs 0.6561386 8.704356e-02 0.40486283 1.0633672  Presence of ID physician 0.5679383 9.539173e-02 0.29203169 1.1045169  Teaching hospital ID fellows 1.6618250 9.003628e-02 0.92357635 2.9901829  Structural ID meetings 1.3597892 3.938322e-01 0.67031807 2.7584317  Mean Glomerular filtration 0.9982758 4.844822e-01 0.99337311 1.0032026  Urine culture positive 1.1821245 2.989992e-01 0.86183480 1.6214457  Feedback on antibiotic  prescription at department  level 0.8864436 6.578648e-01 0.51966015 1.5121081  Feedback on antibiotic  prescription at individual  level 0.8525280 6.920165e-01 0.38677630 1.8791327  **Feedback on antibiotic**  **resistance rates of hospital 2.0450838 1.087730e-02 1.17974635 3.5451414**  Individual advice regarding  streamlining therapy 0.9099905 7.386569e-01 0.52254886 1.5846991  Antibiotic formulary 0.4635769 2.380316e-01 0.12914056 1.6641055  Restrictive use antibiotics 1.0330720 9.314706e-01 0.49169573 2.1705250  Selective reporting of culture  result 1.8725864 5.331769e-02 0.99105158 3.5382414  Automatic stop-order 3.6445014 7.951857e-02 0.85845211 15.4724882  Local resistance rates used  in determining local  guideline 1.0659881 8.991223e-01 0.39636619 2.8668710 |

| QI 7B: Treat UTI in men according to local guideline |
| --- |
| Odds P 95% CI  Department Urology 1.30414543 0.557755280 0.536068706 3.1727189  Age 0.99444704 0.401827490 0.981539027 1.0075248  Admission at night 0.91031379 0.812902428 0.383415041 2.1612903  Comorbidity 1.42578425 0.155801327 0.873336759 2.3276940  Urological comorbidity 0.99519291 0.985107773 0.599563027 1.6518846  Diabetes 1.10216203 0.732000816 0.631169852 1.9246184  Allergy to (any) antibiotic 0.83215893 0.662086477 0.364660471 1.8989952  Antibiotic therapy <14 days 1.01719743 0.932484076 0.685234725 1.5099798  Febrile UTI 0.84216290 0.436219642 0.546189840 1.2985198  Teaching hospital department 3.41356218 0.098744688 0.794242866 14.6710877  Mean % female physicians 0.99036068 0.427395815 0.966925517 1.0143638  Microbiologist and/or ID  physician present at ward  rounds discussing antibiotics 2.25496359 0.191694373 0.664652736 7.6504023  Residents working at department 0.04161095 0.015537833 0.003173958 0.5455241  Structural education on  antibiotics for residents 0.58832179 0.310506187 0.210827115 1.6417363  Structural education on  antibiotics for senior staff  members 3.92987908 0.034094224 1.108474111 13.9326209  **Quality improvement project**  **concerning antibiotics < 3yrs 0.28623141 0.003031846 0.125371261 0.6534864**  Changes in antibiotic  procedures or policies < 3yrs 0.99842923 0.996976012 0.442356654 2.2535231  Presence of ID physician 0.48589645 0.238208677 0.146242298 1.6144123  Teaching hospital ID fellows 1.11741797 0.820783665 0.427068909 2.9237037  Structural ID meetings 3.09554257 0.102345842 0.797202750 12.0200085  Mean Glomerular filtration 0.99853831 0.683088184 0.991211259 1.0059195  Urine culture positive 1.40735088 0.061582668 0.983517051 2.0138304  Feedback on antibiotic  prescription at department  level 0.57144733 0.274062113 0.209447972 1.5591082  Feedback on antibiotic  prescription at individual  level 1.26636246 0.715958179 0.354308170 4.5262120  Feedback on antibiotic  resistance rates of hospital 0.84641176 0.693085724 0.369365033 1.9395796  Individual advice regarding  streamlining therapy 2.87272018 0.027260920 1.126131526 7.3282037  Antibiotic formulary 0.41334344 0.377724090 0.057922420 2.9496833  Restrictive use antibiotics 1.64309930 0.427460156 0.481261560 5.6097880  Automatic stop-order 0.11534474 0.145612700 0.006275785 2.1199593  Local resistance rates used  in determining local  guideline 0.09530590 0.018183972 0.013575181 0.6691045  Selective reporting of culture  result 0.88926884 0.865939609 0.227250314 3.4798591 |
